# Direct Analysis of Pharmaceutical Drugs Using Nano-DESI MS

**DOI:** 10.1155/2016/3591908

**Published:** 2016-09-28

**Authors:** Carlos Cardoso-Palacios, Ingela Lanekoff

**Affiliations:** Department of Chemistry-BMC, Uppsala University, Uppsala, Sweden

## Abstract

Counterfeit pharmaceutical drugs imply an increasing threat to the global public health. It is necessary to have systems to control the products that reach the market and to detect falsified medicines. In this work, molecules in several pharmaceutical tablets were directly analyzed using nanospray desorption electrospray ionization mass spectrometry (nano-DESI MS). Nano-DESI is an ambient surface sampling technique which enables sampling of molecules directly from the surface of the tablets without any sample pretreatment. Both the active pharmaceutical ingredients (APIs) and some excipients were detected in all analyzed tablets. Principal component analysis was used to analyze mass spectral features from different tablets showing strong clustering between tablets with different APIs. The obtained results suggest nano-DESI MS as future tool for forensic analysis to discern APIs present in unknown tablet samples.

## 1. Introduction

Falsifications of pharmaceutical drugs have increased together with the globalization and the worldwide percentage of all medicines which are counterfeit is estimated to be 10% [1, 2]. Counterfeit medicines can harm and kill and cause problems during treatment or recovery of the disease and even result in death. For example, fake vaccines caused 2500 deaths in Nigeria in 1995 [3], and an epidemic of fatal renal failure was a result of paracetamol elixirs containing diethylene glycol [4].

There are systems to prevent and control counterfeiting based on authentication characteristics. Among these technologies are barcodes, holograms, radio-frequency identification, digital watermarks, invisible printing, and chemical and biological tags [5]. Other approaches are mass serialization of the product and working towards a real global trade item number. Track and trace technologies are becoming more advanced, but they need still to be improved continuously and have to be used in a multilevel approach, in order to detect more sophisticated falsifications [5].

For chemical analysis, drugs may be analyzed by presumptive or confirmatory tests. Presumptive tests are typically on-field, fast, and easy to use. Many colorimetric assays, chemical as well as immunological, and thin layer chromatography (TLC) are popular presumptive techniques [6]. Confirmatory tests on the other side are slower but are more selective, precise, and accurate [1]. Techniques for confirmatory tests include different spectroscopy and separation techniques [1, 7–12]. Separation techniques can be coupled to a variety of detection techniques, such as UV-visible detectors, flame ionization detector (FID), and electron capture detector (ECD), or mass spectrometry (MS) which also can provide a fingerprint of molecules present in the sample [13].

Direct MS analysis of tablets is possible using ambient surface sampled ionization techniques such as direct analysis in real-time (DART), desorption electrospray ionization (DESI), or surface desorption atmospheric pressure chemical ionization (DAPCI) [12, 14–17]. The benefit of these techniques is the immediate analysis of the molecular matrix on the tablet without the need for prior sample preparation or dissolution. A new ambient ionization technique for surface sampling is nanospray desorption electrospray ionization (nano-DESI) [18]. Nano-DESI utilizes two fused silica capillaries for extremely localized desorption of molecules from a surface into the continuously flowing liquid bridge between the capillaries. Nano-DESI hyphenated with MS has been employed in different applications such as mass spectrometry imaging (MSI) of molecules in thin tissue sections [19–26], bacterial characterization [27–29], direct analysis of crude petroleum [30, 31], and atmospheric samples [32–38].

Herein, we use nano-DESI MS for the first time to directly analyze fourteen different brands of tablets containing four different APIs, namely, ibuprofen, paracetamol, sildenafil (Viagra-type), or tadalafil (Cialis-type). By use of PCA we show that it is possible to cluster the tablets based on their APIs and their excipients.

## 2. Material and Methods

### 2.1. Tablets and Sample Preparation

Thirteen different brands of tablets and one gel were investigated; three contained ibuprofen, four contained paracetamol, four contained sildenafil, and three contained tadalafil. A table of all investigated tablets, their trade names, and amount API can be found in Table S1 in Supplementary Material available online at http://dx.doi.org/10.1155/2016/3591908. Some of the tablets were obtained from customs after being seized and some were bought fresh. The tablets were prepared by fracturing, which exposed a fresh surface for analysis. The fractured tablet was then manually placed under the nano-DESI probe using a micromanipulator (500 MIM, Quarter Research and Development, Bend, OR).

### 2.2. Nano-DESI MS Analysis

The nano-DESI probe was comprised of two fused silica capillaries (50 *μ*m ID and 150 *μ*m OD, Polymicro Technologies, Molex) positioned at an angle to each other, as previously described [18]. A continuous flowing solvent, propelled at 1 *μ*L/min using a syringe pump (Harvard Apparatus, Holliston, MA), generates a liquid bridge between the two capillaries. As the liquid bridge makes contact with the surface of the tablet, molecules are extracted into the solvent and transported to the inlet of the mass spectrometer for subsequent electrospray ionization and MS analysis. Three solvents were evaluated: methanol and water (9 : 1), methanol and water (7 : 3), and isopropanol and water (9 : 1). As no significant difference between the solvents was found, all analyses were performed using methanol and water (9 : 1). Data was acquired using a Q-Star XL (AB Sciex) in full MS mode, scanning between* m/z* 100 and 2000 with a spray voltage of 3000 V. The interface heater temperature was 200°C, the ion sources gas 1 and 2 (GS1 and GS2) were set to 0 and the curtain gas (CUR) was set to 10.

To continuously trace electrospray stability, an internal standard consisting of lysophosphatidylcholine (LPC) 19 : 0 was included in the nano-DESI solvent to a final concentration of 3 *μ*M. The ions obtained from the standards were* m/z* 104, which is a choline fragment,* m/z* 538, 560, and 576 being LPC 19 : 0 [M + H]^+^, [M + Na]^+^, and [M + K]^+^, respectively, and* m/z* 1075 which is an LPC 19 : 0 dimer.

### 2.3. Principle Component Analysis

Matrices of the acquired data from different tablets and replicates were constructed using the relative abundances of every detected ion. Ions originating from the internal standard, LPC 19 : 0, were excluded. Several technical replicates were obtained from each tablet as shown in Table S2. Data with a total ion current of at least 10^4^ counts per second and, at the minimum, one API or one excipient were included in the data matrix. The matrices were analyzed by principal component analysis (PCA) using The Unscrambler, version 9.7 (CAMO Software AS). The default settings were used, with leverage correction and the unweighted mode. The original variables were reduced to three principal components in the analysis.

## 3. Results and Discussion

**Figure 1:**
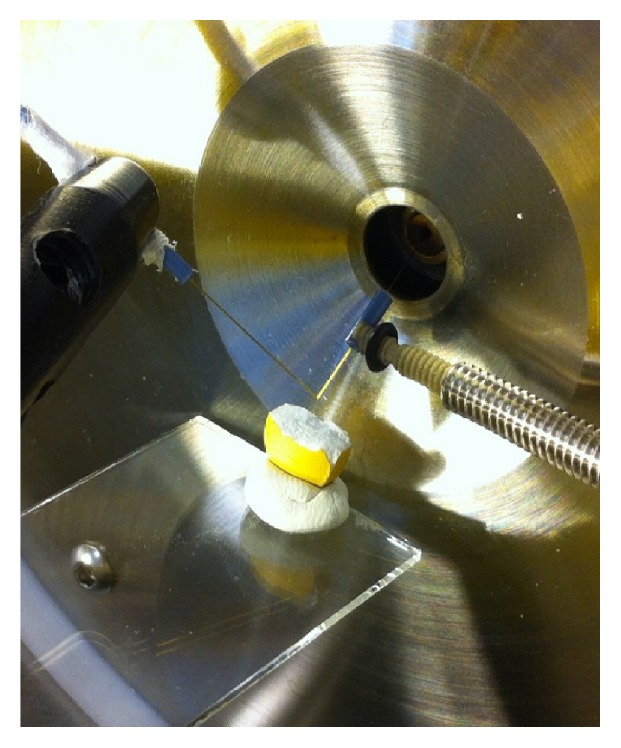
Photograph of the nano-DESI MS setup for online sampling and analysis from the surface of a fractured tablet.

Figure 1 shows a photograph of the nano-DESI MS setup for direct sampling from the tablet surface. The tablet was mounted on a glass slide which was manually moved into position using a micromanipulator. The four APIs could be readily detected with nano-DESI MS by simply dividing the tablet, positioning it under the liquid bridge, and allowing the liquid to come into contact with the surface of the tablet. Molecules from the tablet surface were readily desorbed into the nano-DESI solvent for subsequent electrospray ionization and MS detection.

**Figure 2:**
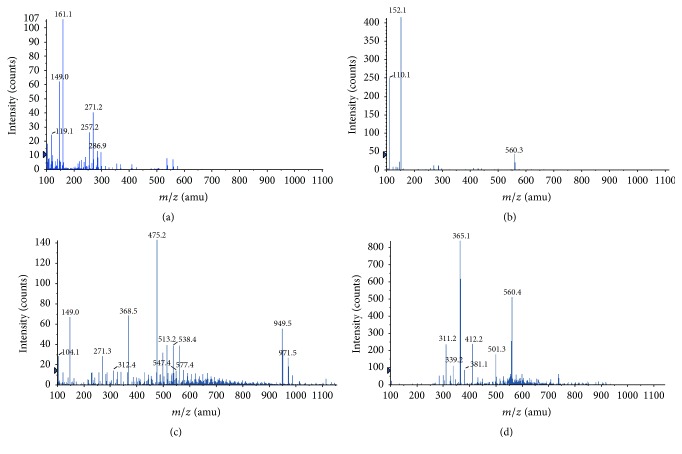
Mass spectra from direct tablet analysis using nano-DESI MS; (a) ibuprofen containing tablet, showing ibuprofen specific fragment ions at* m/z* 119 and 161, (b) paracetamol containing tablet, showing paracetamol specific fragment ions at* m/z* 110 and 152, (c) sildenafil containing tablet, showing specific sildenafil* m/z* 475 ion and* m/z* 513 ion, and (d) tadalafil containing tablet, showing specific tadalafil* m/z* 412 ion and lactose* m/z* 365 ion.

Figures 2(a)–2(d) show four typical mass spectra from four different tablets. The detected ions corresponding to APIs are found in Table 1. The mass spectrum in Figure 2(a) is generated from an ibuprofen containing tablet. Ibuprofen is difficult to detect in positive mode but in this study a low abundant molecular ion was detected at* m/z* 207. Additionally, Figure 2(a) shows peaks at* m/z* 119 and 161 which are attributed as ibuprofen fragment ions, corresponding to C_9_H_11_ and C_12_H_17_, respectively [39]. The mass spectrum in Figure 2(b) is generated from a paracetamol containing tablet. It shows the fragment ion of paracetamol at* m/z* 110, corresponding to C_6_H_8_NO, and protonated paracetamol at* m/z* 152 [40]. Additionally, paracetamol cationized on sodium was detected at low abundance at* m/z* 174.  Figure 2(c) shows the mass spectrum from a sildenafil containing tablet with the peaks at* m/z* 475 and 513 corresponding to protonated sildenafil and sildenafil cationized on potassium, respectively. Similarly, the peaks at* m/z* 949 and 971 correspond to protonated and potassiated sildenafil dimers, respectively. These assignments are in agreement with previously published studies [41–44]. The mass spectrum acquired from a tablet containing tadalafil is shown in Figure 2(d). Peaks corresponding to tadalafil are found at* m/z* 268 and 412, corresponding to a tadalafil fragment ion and the molecular ion of tadalafil cationized on sodium [41, 44, 45]. The signal intensity of tadalafil in Cialis-type tablets was lower than the other APIs analyzed. The signal intensities of APIs were similar between brand name and generic pharmaceutical drugs in all tablets analyzed. Additional, tentatively assigned, ions detected from the tablets and gel using nano-DESI MS were lactose (*m/z* 365) and glucose (*m/z* 203). These have also previously been detected using DESI MS [1]. Lactose is not an excipient in analgesic tablets but is present in both Viagra- and Cialis-type tablets and detected in Cialis-type tablets. A notable exception of the Viagra-type samples was Kamagra which is a gel and contains both lactose and glucose, which are both detected.

**Figure 3:**
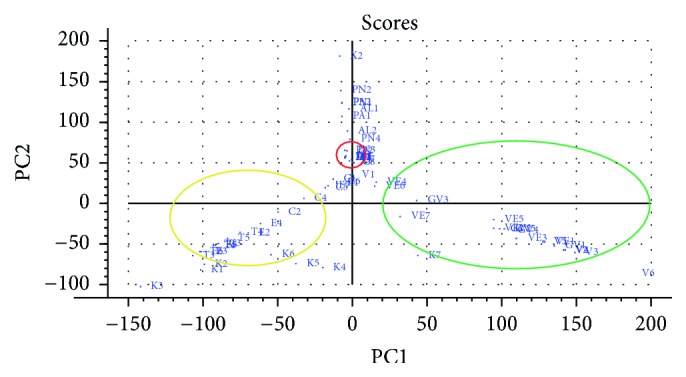
PCA scores of all samples. Matrix consisted of 63 samples and 112 ions. Important ions contributing to the new PCs according to the X-loadings are* m/z* 110, 152, 365, 366, 475, and 476. The red circle shows ibuprofen containing medicines, the yellow circle shows tadalafil containing samples, and the green circle shows Viagra-type tablets.

Mass spectra generated by full-scan analysis of chemical compounds are typically very complex and consist of a large numbers of peaks. To simplify and understand the structure of the data acquired from the tablets, principal component analysis (PCA) was employed [46].  Figure 3 shows the PCA scores for all samples using the two first principle components, PC1 and PC2. The red ring circles the ibuprofen containing tablets which are mainly dependent on PC2. The yellow and green rings circle tadalafil containing tablets and the three different Viagra tablets, respectively. The latter are mainly separated based on PC1. APIs and/or lactose contributed greatly to the X-loadings, especially* m/z* 110, 152, 365, 366, 475, and 476. This highlights the impact of APIs and implies potential problems when using PCA to discriminate between tablets having the same API. However, counterfeit drugs which do not contain the proper API will be easily distinguished. In addition, PCA can distinguish between pills containing different APIs. A high abundance of excipients can, however, cause a different clustering pattern which is not depending on APIs, as in the cases of Viagra and Kamagra. Viagra and Kamagra have the same API, but Kamagra is a gel with high lactose and glucose content which clusters it differently than Viagra as shown in Figure S1. By generating reference libraries of spectra from known tablets, nano-DESI MS in combination with PCA can be a tool to distinguish counterfeit and falsified pharmaceutical drugs.

In conclusion, thirteen different tablets, and one gel, with four different APIs were successfully analyzed with nano-DESI MS and clustered using PCA. The study shows that nano-DESI MS is a useful and powerful technique to detect APIs and excipients without the need for any sample preparation. The method has possibilities of automatization and quantification. No significant differences between seized and generic tablets were detected, but the technique could be further developed by coupling a more sensitive and higher resolving mass spectrometer nano-DESI enabling additional information about the tablet matrix to be obtained. Future perspectives of the use of nano-DESI MS as an anticounterfeit technology are quantification of APIs and excipients and tandem MS/MS analysis for increased accuracies and identifications. Additionally, reference libraries of spectra from known tablets can be used to rapidly find falsified or counterfeit tablets in a quest to limit harmful drugs in society.

## Supplementary Material

Table S1 shows the names and the specifications of the pharmaceutical drugs investigated. In table S2 the number of replicate analysis before and using the different tablets are specified. Figure S1 shows the PCA-scores for sildenafil containing tablets.

## Figures and Tables

**Table 1:** APIs analyzed in this study, molecular formula, molecular weights, molecular structure, and detected *m*/*z* ions with proposed adducts.

API	Detected peaks (*m*/*z*)
Ibuprofen (C_13_H_18_O_2_) 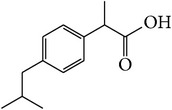 MW = 206,3 g/mole	119 161 207 [M + H]^+^

Paracetamol (C_8_H_9_NO_2_) 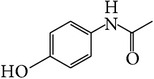 MW = 151,2 g/mole	110, 152 [M + H]^+^ 174 [M + Na]^+^

Sildenafil (C_22_H_30_N_6_O_4_S) 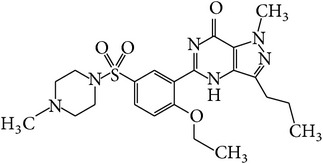 MW = 474,6 g/mole	475 [M + H]^+^ 497 [M + Na]^+^ 513 [M + K]^+^ 949 [2M + H]^+^ 971 [2M + Na]^+^

Tadalafil (C_22_H_19_N_3_O_4_) 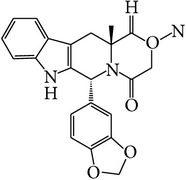 MW = 389,4 g/mole	268 412 [M + Na]^+^
