# Supplementary material for: Direct Analysis of Pharmaceutical Drugs Using Nano-DESI MS
Source: J Anal Methods Chem. 2016 Sep 28;2016:3591908. doi: 10.1155/2016/3591908 (PMC5059531; doi:10.1155/2016/3591908)
Supplement: Supplementary file 1 — Table S1 shows the names and the specifications of the pharmaceutical drugs investigated. In table S2 the number of replicate analysis before and using the different tablets are specified. Figure S1 shows the PCA-scores for sildenafil containing tablets. [file 3591908.f1.pdf]

Direct Analysis of Pharmaceutical Drugs using nano-DESI MS

Carlos Cardoso Palacios[1], Ingela Lanekoff[1,*]

Department of Chemistry – BMC, Uppsala University

Supporting information

Table S1. Pharmaceutical drugs investigated. Table shows APIs and amount per tablet, manufacturing company and commercial type of drug.

**Tradename**	**API (mg)**	**Company**	**Type**
Ipren (IP)	Ibuprofen (400)	McNeil Sweden AB	Brand name
Ibuzin (IZ)	Ibuprofen (400)	Apofri	Generic
Ibuprofen apofri (IA)	Ibuprofen (400)	Apofri	Generic
Alvedon (AL)	Paracetamol (500)	GlaxoSmithKline	Brand name
Panodil (PN)	Paracetamol (500)	GlaxoSmithKline	Generic
Paracetamol apofri (PA)	Paracetamol (500)	Apofri	Generic
Alvedon (expired) (X)	Paracetamol (500)	AstraZeneca	Brand name
Viagra (V)	Sildenafil citrate (100)	Pfizer	Brand name
Viagra (expired) (GV)	Sildenafil citrate (100)	Pfizer	Brand name
Viagire (VE)	Sildenafil citrate (50)	Roid +	Generic
Kamagra (gel) (K)	Sildenafil citrate (100)	Ajanta Pharma	Generic
Cialis (C)	Tadalafil (20)	Lilly	Brand name
Tadacip (T)	Tadalafil (20)	Cipla	Generic
Erectalis (E)	Tadalafil (20)	Cipla	Generic

Table S2. Number of replicate analysis for each pharmaceutical drug and number of ions chemometrically analyzed with PCA.

Tradename	Replicates
Ipren (IP)	3
Ibuzin (IZ)	2
Ibuprofen apofri (IA)	2
Alvedon (AL)	2
Panodil (PN)	4
Paracetamol apofri (PA)	2
Alvedon (expired) (X)	2
Viagra (V)	6
Viagra (expired) (GV)	5
Viagire (VE)	7
Kamagra (gel) (K)	7
Cialis (C)	8
Tadacip (T)	6
Erectalis (E)	7

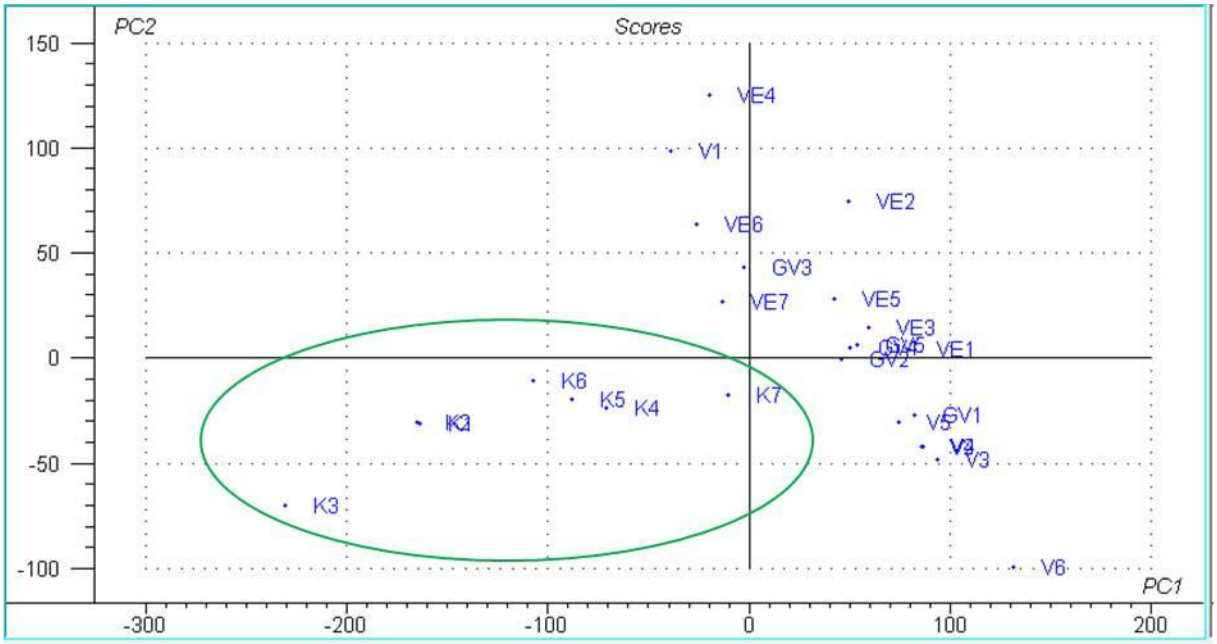

Figure S1. PCA-scores of sildenafil containing tablets. The green ring circles the cluster of Kamagra which is separated from Viagra-type tablets.
